# Hemostatic risk factors in patients with coronary artery disease and type 2 diabetes - a two year follow-up of 243 patients

**DOI:** 10.1186/1475-2840-8-48

**Published:** 2009-09-07

**Authors:** Thomas W Jax, Ansgar J Peters, Gunnar Plehn, Frank-Chris Schoebel

**Affiliations:** 1 Medizinische Klinik und Poliklinik B, Klinik für Kardiologie, Pneumologie und Angiologie, Heinrich-Heine-Universität, Moorenstr. 5, 40225 Duesseldorf, Germany; 2 Profil Institut für Stoffwechselforschung, Hellersbergstrasse 9, 41460 Neuss, Germany; 3 Medizinische Klinik 3, Klinik für Kardiologie, Herzzentrum Wuppertal, and Institut für Herz- und Kreislaufforschung, Dortmund, both Universität Witten/Herdecke, Germany; 4 Medizinische Klinik II, Marienhospital Herne, Ruhr-Universität Bochum, Herne, Germany

## Abstract

**Backgound:**

Thrombosis is regarded to be a key factor in the development of acute coronary syndromes in patients with coronary artery disease (CAD). We hypothesize, that hemostatic and rheological risk factors may be of major relevance for the incidence and the risk stratification of these patients.

**Methods:**

In 243 patients with coronary artery disease and stable angina pectoris parameters of metabolism, hemostasis, blood rheology and endogenous fibrinolysis were assessed. Patients were prospectively followed for 2 years in respect to elective revascularizations and acute coronary syndromes.

**Results:**

During follow-up 88 patients presented with cardiac events, 22 of those were admitted to the hospital because of acute events, 5 Patients were excluded due to non- cardiac death. Patients with clinical events were found to be more frequently diabetic and presented with a more progressed coronary atherosclerosis. Even though patients with diabetes mellitus demonstrated a comparable level of multivessel disease (71% vs. 70%) the rate of elective revascularization was higher (41% vs. 28%, p < 0.05). The results were also unfavorable for the incidence of acute cardiovascular events (18% vs. 8%, p < 0.01). In comparison to non-diabetic patients diabetics demonstrated significantly elevated levels of fibrinogen (352 ± 76 vs. 312 ± 64 mg/dl, p < 0.01), plasma viscosity (1.38 ± 0.23 vs. 1.31 ± 0.16 mPas, p < 0.01), red blood cell aggregation (13.2 ± 2.5 vs. 12.1 ± 3.1 E, p < 0.05) and plasmin-activator-inhibitor (6.11 ± 3.4 vs. 4.7 ± 2.7 U/l, p < 0.05).

**Conclusion:**

Pathological alterations of fibrinogen, blood rheology and plasminogen-activator-inhibtor as indicators of a procoagulant state are of major relevance for the short-term incidence of cardiac events, especially in patients with diabetes mellitus type 2, and may be used to stratify patients to specific therapies.

## Introduction

Apart from established, traditional cardiovascular risk factors like e.g. hypercholesterolemia, parameters of hemostasis, endogenous fibrinolysis and platelet activity are more and more recognized of being of importance in the pathogenesis of atherosclerosis and acute coronary syndromes especially in diabetic patients [1-9].

A recent study could show a diabetic specific coronary morphology that is strongly related to hemostasis and rheology [10]. Both parameters of endogenous fibrinolysis and markers of platelet activity showed a relevant association on the extent of coronary sclerosis. The metabolic changes with consecutive dysbalances of platelet activity, hemostasis and endogenous fibrinolysis, and rheology favor a prothrombotic milieu. These systemic changes may result in an increased, thrombosis driven atherosclerotic process [11], in part explaining the advanced coronary plaque burden and in particular the more distal pronounced distribution of coronary atherosclerosis in patients with diabetes mellitus.

The potential role of these parameters for cardiovascular events was shown in large epidemiological studies [12]. Coronary thrombosis is thought to play a major role for the pathogenesis of acute coronary syndromes [13]. Therefore parameters indicating and predisposing arterial thrombosis might be of major relevance for the higher rate of cardiovascular events in patients with diabetes mellitus.

The present study characterizes the cardiovascular risk profile of 243 patients evaluating the influence on the occurrence of cardiovascular events. Besides of traditional cardiovascular risk factors, parameters of hemostasis, endogenous fibrinolysis and blood rheology were assessed.

## Methods

The study included a total of 264 consecutive patients with CAD undergoing elective coronary angiography either for suspected CAD or with suspected progression of known CAD. Key exclusion criteria were unstable angina pectoris, myocardial infarction within the last two months, acute or chronic heart failure (NYHAIII or IV), severe chronic and acute infection and malignant tumors.

With the beginning of the study the patients gave their informed consent to additional blood sampling during the routine investigation. The patients were informed in detail about the aims of the study and that a follow up was planned. After two years telephone interviews were carried out. If patients could not be contacted by telephone or at their addresses, their relatives or their general practitioners were contacted. As dependent variables for the follow-up period of two years the occurrence of the following events were examined: unstable angina pectoris, myocardial infarction, cardiac death, non- cardiac death, elective PTCA or ACB operation and acute cardiovascular revascularization. In case of present unstable angina pectoris, stable but severe functional limitations of patients or an inadequate medication, the interviewer contacted the general practitioner for further information.

### Definition of the cardiovascular risk profil

Hypercholesterolemia was presupposed with total cholesterol above 220 mg/dl, with a positive history of hypercholesterolemia and/or with lipid reducing therapies. The same was assumed for hypertriglyceridemia above 200 mg/dl. Thus dyslipoproteinemia was diagnosed when hypercholesterolemia or hypertriglyceridemia occured. Hypertension was defined in patients with a resting blood pressure above systolic 140 mmHg or diastolic 90 mmHg, with a history of hypertension or with antihypertensive medication. Nicotin consumption was assessed by history. Diabetes was defined by fasting plasma glucose concentration above 126 mg/dl or an antidiabetic therapy. Recent studies suggest, that a high percentage of cardiac patients present with newly diagnosed type 2 diabetes by use of oral glucose tolerance tests (OGTT) [14-16]. OGTT was not part of this protocol; therefore the true rate of diabetes may be underestimated.

### Coronary Angiography

In all patients coronary angiograms with imaging of the coronary arteries in different standardized projections were carried out. The degree of coronary artery stenosis was assessed by two experienced, independent examiners. The severity of CAD was defined as coronary 1,2 or 3 vessel disease corresponding to the number of affected main vessels with hemodynamic relevant stenosis above 50%. Extent of coronary sclerosis was determined by a special grading system in accordance with an evaluation system of the American Heart Association [17]. Based on these findings the coronary artery system was divided into 15 segments. The segment with a higher degree of stenosis got a higher score. The sum of these scores was divided by the number of judged segments. The result presents the extent of coronary atherosclerosis. To get information about the distribution pattern of coronary sclerosis a proximal score was calculated from the proximal segments and a distal score was calculated from the distal segments of the coronary arteries and of first degree branches.

### Laboratory methods

Blood samples for the different studies were taken at the day of cardiac catheterization between 7:00 and 8:00 am according to a standardized protocol. Before venous puncture was performed patients had spent a rest period in lying position for 30 minutes. Venous puncture of an antecubital vene was done with a butterfly canula after a maximum venous congestion time of 30 seconds and a maximum congestion pressure of 40 mmHg. After decompression the first 4 ml of blood were not used. Blood samples were sampled in vacutainer tubes. Samples were frozen at a temperature of minus 80°C. For parameters of hemostasis and endogenous fibrinolysis tubes with 0.129 M Na_2 _citrat were used. Determination of plasma viscosity and red blood cell aggregation was performed from EDTA tubes within 4 hours after sample taking. The samples were analyzed immediately after preparation or from deeply frozen plasma within three months after sample taking.

Plasma fibrinogen levels were determined according to method described by Clauss [18]. Plasmaviscosity was determined by capillary tube viscosimeter of Rheomed, Aachen (Germany). For determination of red blood cell aggregation an erythrocyte aggregometer (Myrenne, Roetgen, Germany) according to the method of Schmid-Schönbein [19] was used. Plasma tissue plasminogenactivator antigen concentration was measured by enzymimmunoassay. Determination of Plasminogenactivator inhibitor activity was performed by chromogenic enzymassay.

### Statistical methods

Statistical analyses were performed using SPSS software (Statistical Package for Social Sciences for Windows; SPSS 12, Munich). The results were tested for normal distribution by Kolmogoroff-Smirnoff test. For normally distributed parameters group comparison was made by use of paired and unpaired Student's t-test. Mann-Whitney U-Test was used for non-normally distributed parameters. The Pearson correlation coefficient or the Spearman's rank correlations were used to describe the association between the variables in the study. Chi square test was used for comparison of non continuous data. Statistical significance was defined as p < 0.05.

## Results

**Figure 1:**
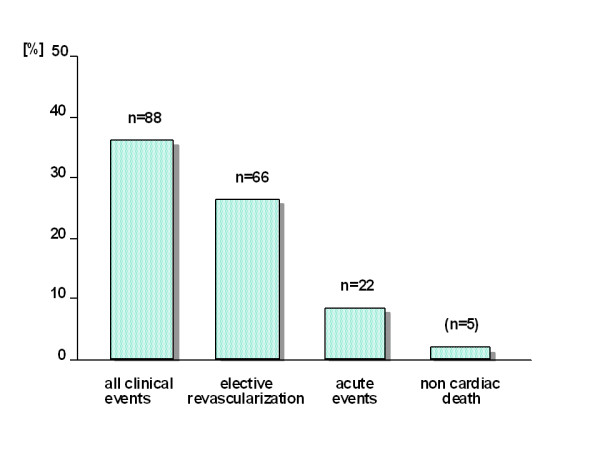
**clinical events in 243 patients**. Elective revascularizations include aorto-coronary bypass operation and angioplasty, acute events include unstable angina pectoris, acute myocardial infarction and cardiac death.

Out of 264 originally selected patients a clinical course after 2 years could be documented in 243 patients. Thus the feed back rate was 91.3%. Patient characteristics are given in table 1. Per definition in 150 patients there was no cardiovascular event. Three patients died from non- cardiac disease. Within the follow up period 88 patients developed a cardiovascular event. 66 of them underwent elective revascularization (figure 1). Of those 44 underwent PTCA and 22 ACB bypass surgery. 22 patients suffered from an acute cardiovascular event: 11 from an unstable angina pectoris, 8 from an acute myocardial infarction and 3 from a cardiac death. Related to the observed group (n = 241) these figures correspond to an elective rate of revascularization of 27,3% (PTCA, ACB) and to an acute rate of events of 9,1%.

**Table 1:** patient characteristics

	clinical event	without clinical event	
	n = 88	n = 150	p - value
age (in years)	58 ± 10	56 ± 11	n.s.
Male (%)	85	84	n.s.
			
*risk profile*			
hypertension (%)	59	52	n.s.
diabetes mellitus	31	11	<0,001
hyperlipidemia (%)	78	78	n.s.
hypercholesterinemia (%)	69	70	n.s.
hypertriglyceridemia (%)	29	32	n.s.
history of smoking or smoking (%)	69	66	n.s.
positive family history (%)	44	42	n.s.
			
*Anamnese*			
history of myokardial infarction (%)	44	51	n.s.
history of ACB operation (%)	10	5,4	n.s.
history of PTCA (%)	57	61	n.s.
			
*Medication*			
nitrates (%)	87	80	n.s.
calcium channel blockers (%)	50	41	n.s.
betablockers (%)	41	46	n.s.
ace-inhibitors (%)	31	28	n.s.
thrombocytaggregationinhibitors (%)	73	78	n.s.
lipid lowering medication (%)	37	45	n.s.
insuline (%)	1,1	1,4	n.s.
oral andiabetic medication (%)	10	3,4	p < 0,05

In regard to the characterization of patients those with coronary ischemic event differed from those without an event by a higher share of diabetic patients (see table 1).

### Severity and extend of CAD

In assessing the severity and extend of coronary sclerosis it became clear that there was a bigger share of coronary multiple vessel disease (72%) in the group with clinical event than in the group without clinical event (49%). These results showed that the increased severity of CAD also resulted in a higher extend of coronary sclerosis which was reflected in proximal as well as distal distribution of coronary sclerosis (table 2)

**Table 2:** severity and extent of coronary atherosclerosis

	clinical event	without clinical event	p-value
	n = 88	n = 150	
*severity of coronary artery disease*			
1- vessel disease (%)	28	51	
2 - vessel disease (%)	36	28	
3 - vessel disease (%)	36	22	p < 0,01
			
*extent of coronary atherosclerosis*			
total score	1,67 ± 0,71	1,31 ± 0,73	p < 0,001
proximal score	1,83 ± 0,74	1,49 ± 0,77	p < 0,001
distal score	1,43 ± 0,79	1,03 ± 0,70	p < 0,001

### Metabolic risk factors

There was no significant difference between the two groups with regard to metabolic risk factors. Only *blood glucose *discriminated which can be explained by the high prevalence of diabetic patients with a clinical event in this group (table 3).

**Table 3:** parameters of metabolism, hemostasis endogenous fibrinolysis and blood rheology

	clinical event	without clinical event	p-value
	n = 88	n = 150	
total cholesterol (mg/dl)	243 ± 49	238 ± 44	n.s.
hdl - cholesterol(mg/dl)	44 ± 11	45 ± 12	n.s.
ldl - cholesterol(mg/dl)	182 ± 49	173 ± 41	n.s.
triglycerides (mg/dl)	209 ± 143	185 ± 113	n.s.
lipoproteine (a) (mg/dl)	32 ± 39	34 ± 41	n.s.
uric acid (mg/dl)	5,8 ± 1,6	6,6 ± 1,7	n.s.
glucose (mg/dl)	108 ± 51	92 ± 35	<0,05
			
tissue -plasminogenactivator (μg/l)	9,3 ± 3,5	8,2 ± 3,0	<0,05
plasminogenaktivator-inhibitore (U/ml)	4,9 ± 2,5	4,9 ± 3,0	n.s.
			
fibrinogen (mg/dl)	330 ± 65	319 ± 70	n.s.
plasma viscosity (mPa × s^-1^)	1,3 ± 0,1	1,3 ± 0,2	n.s.
red blood cell aggregation M (E)	7,7 ± 2,4	7,5 ± 2,3	n.s.
red blood cell aggregation M1 (E)	12,4 ± 2,9	12,3 ± 3,0	n.s.
hematocrit (%)	43,6 ± 3,2	43,1 ± 3,1	n.s.

### Parameter of hemostasis and blood rheology

No difference could be found between the two groups with regard to fibrinogen levels and rheological parameters. Parameters of endogeneous fibrinolysis showed a significant increase of tissue-plasminogen-activator in those patients who suffered from a clinical event in the follow up period (table 3).

### Patients with diabetes mellitus

**Figure 2:**
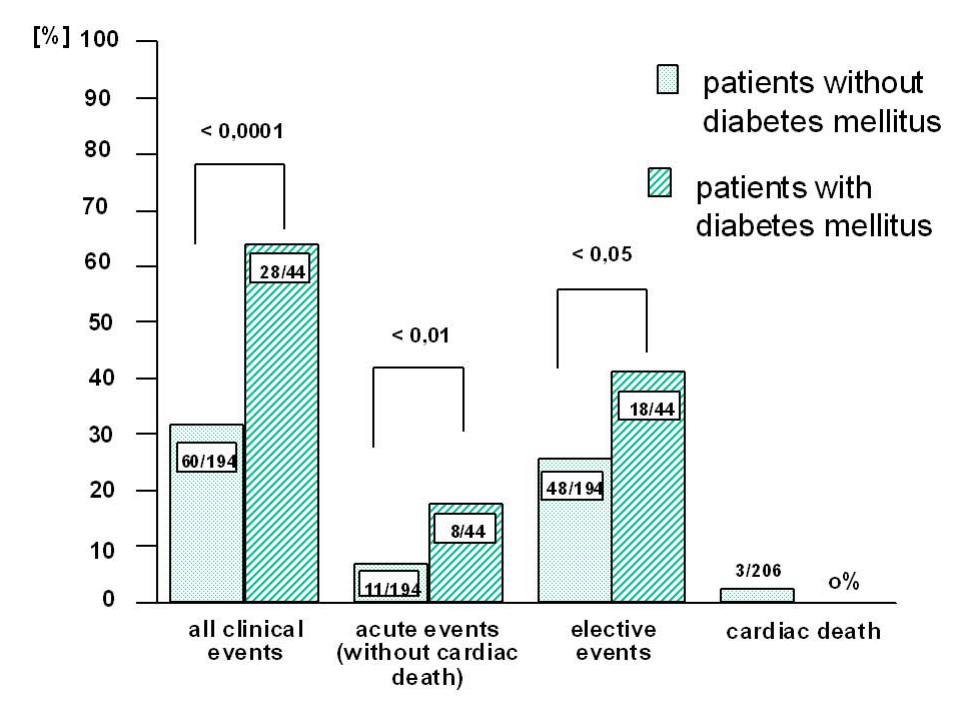
**clinical events for patients with and without diabetes mellitus**. Elective revascularizations include aorto-coronary bypass operation and angioplasty, acute events include unstable angina pectoris, acute myocardial infarction and cardiac death.

Cardiovascular events were significantly higher in the group of diabetic patients with 64% compared to 31% in the non- diabetic patients (figure 2). Even though patients with DM demonstrated a comparable level of multivessel disease in comparison to controls (71% vs. 70%) the rate of elective revascularization was higher (41% vs. 28%, p < 0.05). The share of elective revascularization procedures was higher in the group of nondiabetic patients, if only the patients with an event were calculated (80% vs. 64.3%, p < 0.05). Three cardiac deaths in the non- diabetic group and no death in the diabetic patients did not show significance. The results were also unfavorable for the incidence of acute cardiovascular events (18.3% vs. 28.6%, p < 0.01).

### Metabolic and hemostatic parameters

**Figure 3:**
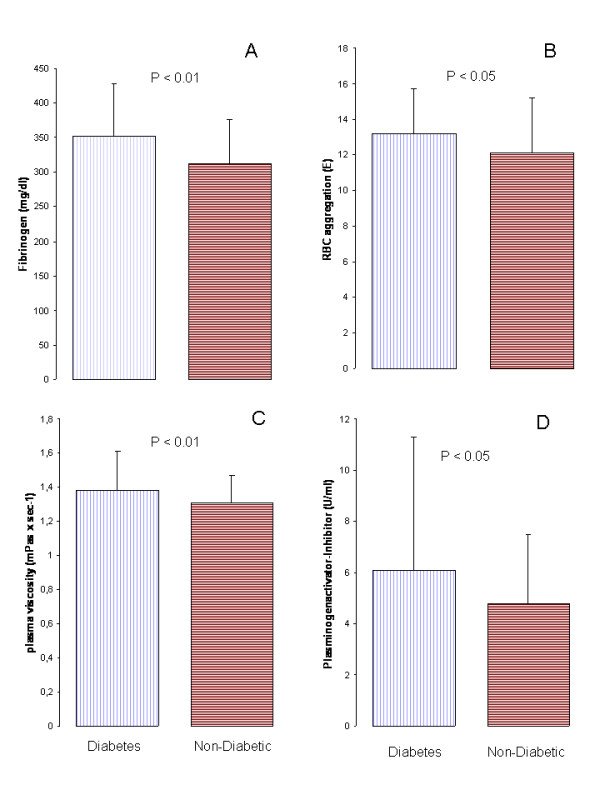
**Rheology in diabetic patients**. Between Patients with and without type 2 diabetes mellitus significant differences were found for fibrinogen (**A**), red blood cell (RBC) aggregation (**B**), plasma viscosity (**C**) and Plasminogenactivator-Inhibitor (PAI) (**D**).

Due to a significant higher rate of cardiovascular events in diabetic patients we further evaluated the metabolic and hemostatic differences between patients with and without diabetes mellitus (table 4 and figure 3). Diabetic patients presented with lower HDL and higher LDL and triglyceride- levels (see table 4). We did not assess long-term metabolic control with HbA1c. Although tissue- plasminogenactivator was comparable between the 2 groups, plasminogen- activator- inhibitor was higher in diabetic patients. Parameters of blood rheology were all elevated in the group of patients with diabetes mellitus (see figure 3).

**Table 4:** parameters of metabolism, hemostasis, endogenous fibrinolysis and blood rheology for patients with and without diabetes mellitus

	diabetes mellitus	non-diabetic patients	p-value
	n = 44	n = 194	
total cholesterol (mg/dl)	254 ± 69	239 ± 43	n.s.
hdl - cholesterol(mg/dl)	42 ± 13	45 ± 12	<0,05
ldl - cholesterol(mg/dl)	188 ± 55	173 ± 43	<0,05
triglycerides (mg/dl)	257 ± 204	183 ± 103	<0,05
lipoproteine (a) (mg/dl)	22 ± 33	36 ± 42	<0,05
glucose (mg/dl)	157 ± 67	88 ± 12	<0,0001
			
tissue -plasminogenactivator (μg/l)	8,9 ± 3,7	8,4 ± 3,0	n.s.
plasminogenaktivator-inhibitore (U/ml)	6,1 ± 5,2	4,8 ± 2,7	<0,05
			
fibrinogen (mg/dl)	351 ± 76	312 ± 64	<0,01
plasma viscosity (mPa × s^-1^)	1,38 ± 0,23	1,31 ± 0,16	<0,01
red blood cell aggregation M (E)	8,7 ± 2,1	7,3 ± 2,0	<0,05
red blood cell aggregation M1 (E)	13,2 ± 2,5	12,1 ± 3,1	<0,05

## Discussion

The presented results on the influence of rheological parameters and factors of endogenous fibrinolysis on the rate of clinical events showed that t-PA concentration is of predictive importance within a follow up period of two years. As the clinical rate of events in this study is essentially determined by elective revascularisation the increase of tPa in our study does not reflect so much the influence on acute clinical events but points to the correlation between tPa and severity and extend of coronary sclerosis, respectively. The incidence of acute coronary events was 9.1% within the two year- period and thus roughly corresponds to the estimated figure of 2-5% per year. Considering the share of patients with coronary multiple vessel disease of 57% the rate of revascularization with 27.3% is representative.

A disturbed endogenous fibrinolysis in diabetic patients has been described in the past [4,9]. The proportion of diabetics in the group with clinical events was relatively high. The increase of t-PA can be judged by an elevation of tPa - tPa inhibitor complexes and a consequently reduced fibrinolysis due to an increase in tPa- inhibitor concentration [12]. In addition, a high extend of atherosclerosis in coronary arteries and other vessel areas reflects a thrombogenic stimulus inducing a secondary, enhanced fibrinolytic activity. In a prior study from our group a parallel increase of t-PA and PAP complexes was measured and thus proved that an elevation of t-PA can reflect an increased fibrinolytic activity [20]. Factors of blood rheology had no predictive significance regarding the incidence of cardiovascular events. This may be explained by the fact that the described patients formed a group where coronary artery disease was manifest while the majority of epidemiological investigations on the prospective importance of fibrinogen were based on patients without any evidence of coronary artery disease [21]. Moreover the period of observation may have been to short and the clinical rate of event to small to give a valid statement.

**Figure 4:**
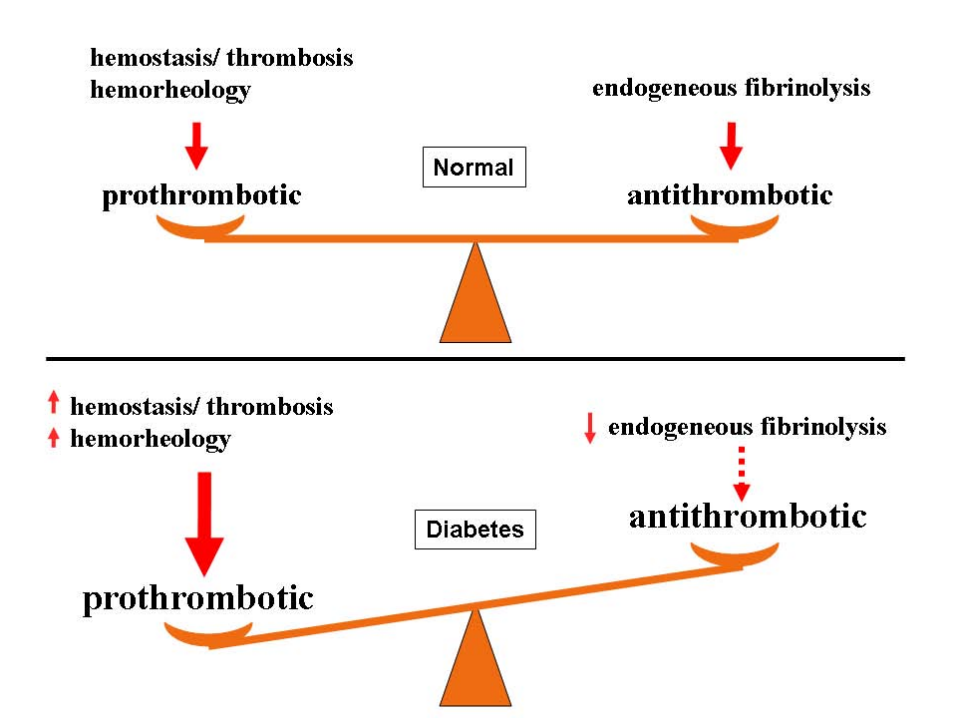
**Balance and dysbalance of hemostasis in diabetes**.

Based on different prospective studies especially fibrinogen is considered as a proven hemostatic factor for an increased cardiovascular risk. A metaanalysis demonstrated a 2.3 fold elevated cardiovascular risk for fibrinogen levels above 320 mg/dl [21]. In addition to an impairment of hemostasis, blood rheology and platelet reactivity and the consequent imbalance of the hemostatic system (figure 4); fibrinogen may directly promote atherogenesis [21].

Apart from patients with hyperfibrinogenemia there were also patients with elevated t-PA and von Willebrand factor with an increased rate of cardiovascular events affected [12]. Other studies also found an elevated t-PA concentration as a risk factor of increased incidence of cardiovascular events. Data from the Physicians Health Study showed a 2.8 fold increased risk between lowest and highest quintile of t-PA concentration [22]. The importance of PAI is underlined by a follow up study of men after primary myocardial infarction. Those patients who showed elevated PAI concentrations as a sign of reduced fibrinolysis were at increased risk of reinfarction [23,24]. High concentrations of von Willebrand factor antigen are supposed to be markers of endothelial lesions and present a procoagulative mechanism due to an inhanced platelet adhesion [25].

The subset of patients with diabetes mellitus showed a marked increase in the number of cardiovascular events, favoring especially the number of acute events. This is in correspondence to the literature, which reports also a higher incidence of acute cardiovascular events like acute myocardial infarction and cardiac death [26] and also a higher incidence of revascularization procedures [27]. The potential role of insulin in atherogenesis has been discussed for 25 years [28]. Results of various prospective studies indicated that elevated plasma insulin levels cause and enhance atherosclerosis [29-31]. Diverse diseases associated with hyperinsulinemia like the metaboliv syndrome, hypertension, adipositas and hypertriglyceridemia show an early and extended manifestation of atherosclerosis [32-37]. Several studies proved that elevated insulin levels are accompanied by reduced endogenous fibrinolysis [1-3,5,7]. On the one hand hyperinsulinemia is associated with a decreased t-PA concentration [5], on the other hand it causes an increase of PAI activity [1-3,7].

Rheological properties in general are a major factor determining microcirculatory function and blood flow. Plasma viscosity and red blood cell aggregation are decisive rheological parameters of microcirculation. Erythrocyte aggregation is described to be closely linked to fibrinogen and alpha2- macroglobulin [38]. A fibrinogen mediated increase of both is not relevant in non- obstructed epicardial arteries under normal circumstances. Impaired blood rheology can restrict coronary blood flow and cause critically myocardial oxygen and nutrition supply in the area of poststenotic microcirculation of successive multiple linked stenoses and collateralized occlusions with heavily impaired vasomotion [39]. Diabetic patients present with impaired hemorheology, as also shown in this study. Patients with diabetes mellitus and stroke have more often an impaired hemorheology as non-diabetic patients [40]. Secondary microcirculatory complications are also associated with higher erythrocyte aggregation and viscosity [41].

Hemorheological disorders are strongly related to metabolic control (HbA1c), lipid parameters and hyperinsulinemia [42,43]. Red blood cell deformability is mediated by nitric oxide, as well as red blood cell aggregation [44]. Nitric oxide is well known to be markedly reduced in patients with type 2 diabetes mellitus and well accepted as a marker of endothelial function. Thus, the rheological impairment of diabetic patients is related to endothelial dysfunction and a possible cause of both micro- and macrovascular complications.

## Conclusion and Future Perspective

In summary, pathological alterations of fibrinogen, blood rheology and plasminogen-activator-inhibtor as indicators of a procoagulant state (figure 4) are of relevance for the short-term incidence of cardiac events, especially in patients with diabetes mellitus type 2. Diabetes predisposes to those multiple alterations, which in turn can have multiple effects on atherosclerosis, vascular morphology and cardiovascular events. The possible link between endothelial dysfunction, red blood cell function and pathological rheology and hemostasis may, in part, connect micro- and macrovascular complications with diabetes mellitus. More research is needed to elucidate these potential mechanisms and possibly develop a tailored therapy for these diabetes specific mechanisms of atherothrombotic disease.

## Competing interests

The authors declare that they have no competing interests.

## Authors' contributions

TJ participated in the design and coordinated the study, performed the statistical analysis and drafted the manuscript. AP coordinated the study, and drafted the manuscript. GP coordinated the study and drafted the manuscript. FS participated in the design and coordinated the study, and drafted the manuscript. All authors read and approved the final manuscript.
